# 4-Aryl-4*H*-naphthopyrans derivatives: one-pot synthesis, evaluation of Src kinase inhibitory and anti-proliferative activities

**DOI:** 10.1186/2008-2231-20-100

**Published:** 2012-12-26

**Authors:** Ali Rafinejad, Asal Fallah-Tafti, Rakesh Tiwari, Amir Nasrolahi Shirazi, Deendayal Mandal, Abbas Shafiee, Keykavous Parang, Alireza Foroumadi, Tahmineh Akbarzadeh

**Affiliations:** 1 Department of Medicinal Chemistry, Faculty of Pharmacy and Drug Design & Development Research Center, Tehran University of Medical Sciences, Tehran, Iran; 2 Department of Biomedical and Pharmaceutical Sciences, College of Pharmacy, The University of Rhode Island, Kingston, RI, USA

**Keywords:** Anticancer activity, Carbonitrile, Naphthopyrans, Protein kinase, Src kinase

## Abstract

**Background:**

A series of 2-amino-4-aryl-4*H*-benzo[h or f]chromene-3-carbonitrile derivatives were synthesized and evaluated for inhibition of Src kinase and cell proliferation in breast carcinoma (BT-20) cell lines.

**Methods:**

The one-pot, three-component reaction of α or β-naphthol, malonitrile and an aromatic aldehyde in the presence of diammonium hydrogen phosphate was afforded the corresponding 2-amino-4-aryl-4*H*-benzo[h or f]chromene-3-carbonitrile derivatives, All target compounds were evaluated for inhibition of Src kinase and cell proliferation in breast carcinoma (BT-20) cell lines.

**Results:**

Among all tested compounds, unsubstituted 4-phenyl analog **4a** showed Src kinas inhibitory effect with IC_50_ value of 28.1 μM and was the most potent compound in this series. In general, the compounds were moderately active against BT-20. 3-Nitro-phenyl **4e** and 3-pyridinyl **4h** derivatives inhibited the cell proliferation of BT-20 cells by 33% and 31.5%, respectively, and found to be more potent compared to doxorubicin (25% inhibition of cell growth).

**Conclusion:**

The data indicate that 4-aryl-4*H*-naphthopyrans scaffold has the potential to be optimized further for designing more potent Src kinase inhibitors and/or anticancer lead compounds.

## Introduction

c-Src kinase is one of the most widely studied member of the Src (acronym of sarcoma) family of non-receptor protein tyrosine kinases (PTKs). c-Src acts as a critical component in a number of signaling pathways that control cell growth, proliferation, invasion, and apoptosis [1-3]. Previous studies have shown that c-Src kinase is highly regulated and is active only at low levels in most normal cells while it is upregulated in many human cancers [4,5]. Most recently emerging data reveals that the predominant consequences of increased c-Src activity in tumor cells results in reduction of cell adhesion, facilitation of motility, and thereby promotion of an invasive phenotype [6]. Interest in designing c-Src kinase inhibitors as a treatment for cancer and in particular, as an antiinvasion strategy has been increased in the last decade. The current popularity of Src kinases as drug targets is due to several factors including the positive correlation between the development of cancer and the upregulation of Src activity and reduction of cancer progression by inhibition of Src kinase in several types of cancer [7-10].

**Figure 1:**
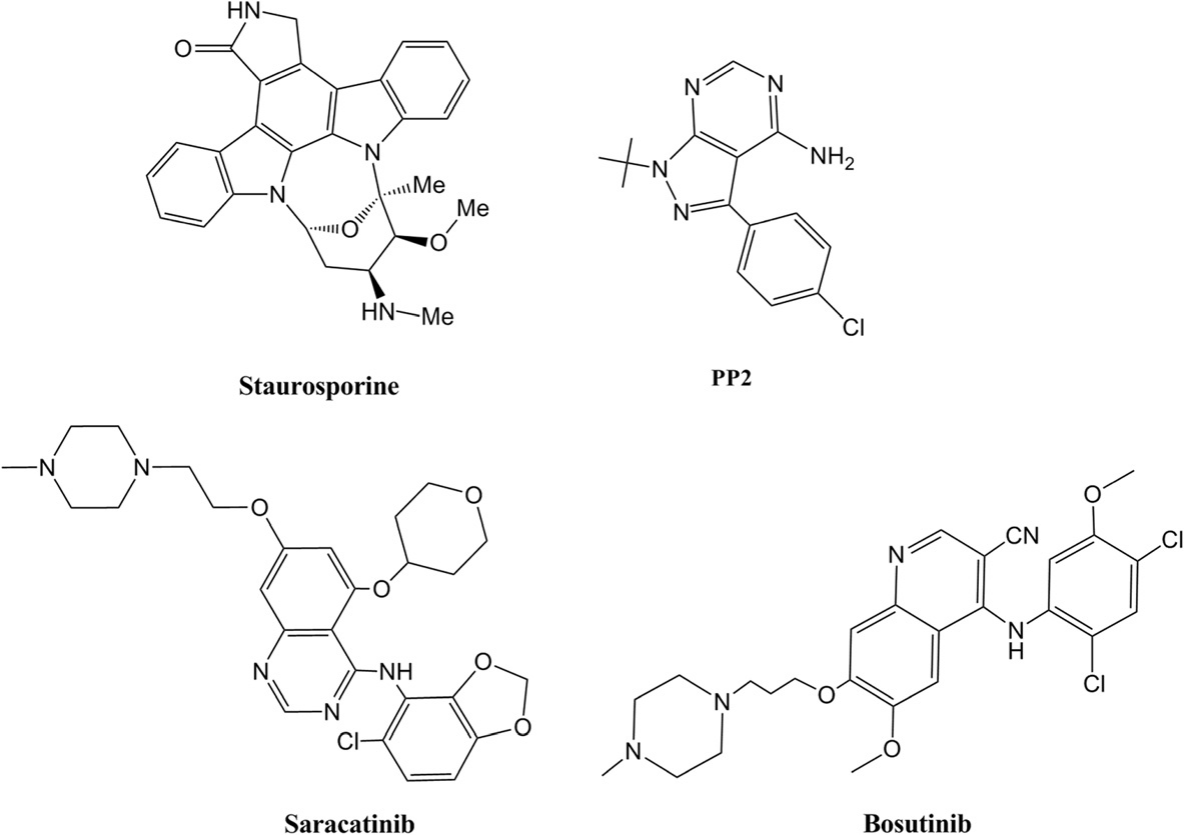
Chemical structures of some of the Src kinase inhibitors.

Several c-Src kinase inhibitors have been identified over the years [11]. Src kinase inhibitor scaffolds span a variety of structural classes, including staurosporine as a standard nonselective protein kinase inhibitor, pyrazolopyrimidine (e.g., PP1, PP2) [12,13], anilinoquinazoline [14,15], and quinolinecarbonitrile [16-18] derivatives. The Saracatinib (AZD0530) [15,19] and Bosutinib (SKI-606) [20,21] with anilinoquinazoline and 3-quinolinecarbonitrile cores, respectively (Figure 1), are among potent Src kinase inhibitors, which are currently in clinical development for the treatment of a wide range of tumor types. Since ATP binding region is strongly conserved among protein kinases, designing selective Src kinase inhibitors is still a great challenge. Therefore, research is needed to explore new scaffolds which selectively inhibit Src kinase domain.

**Figure 2:**
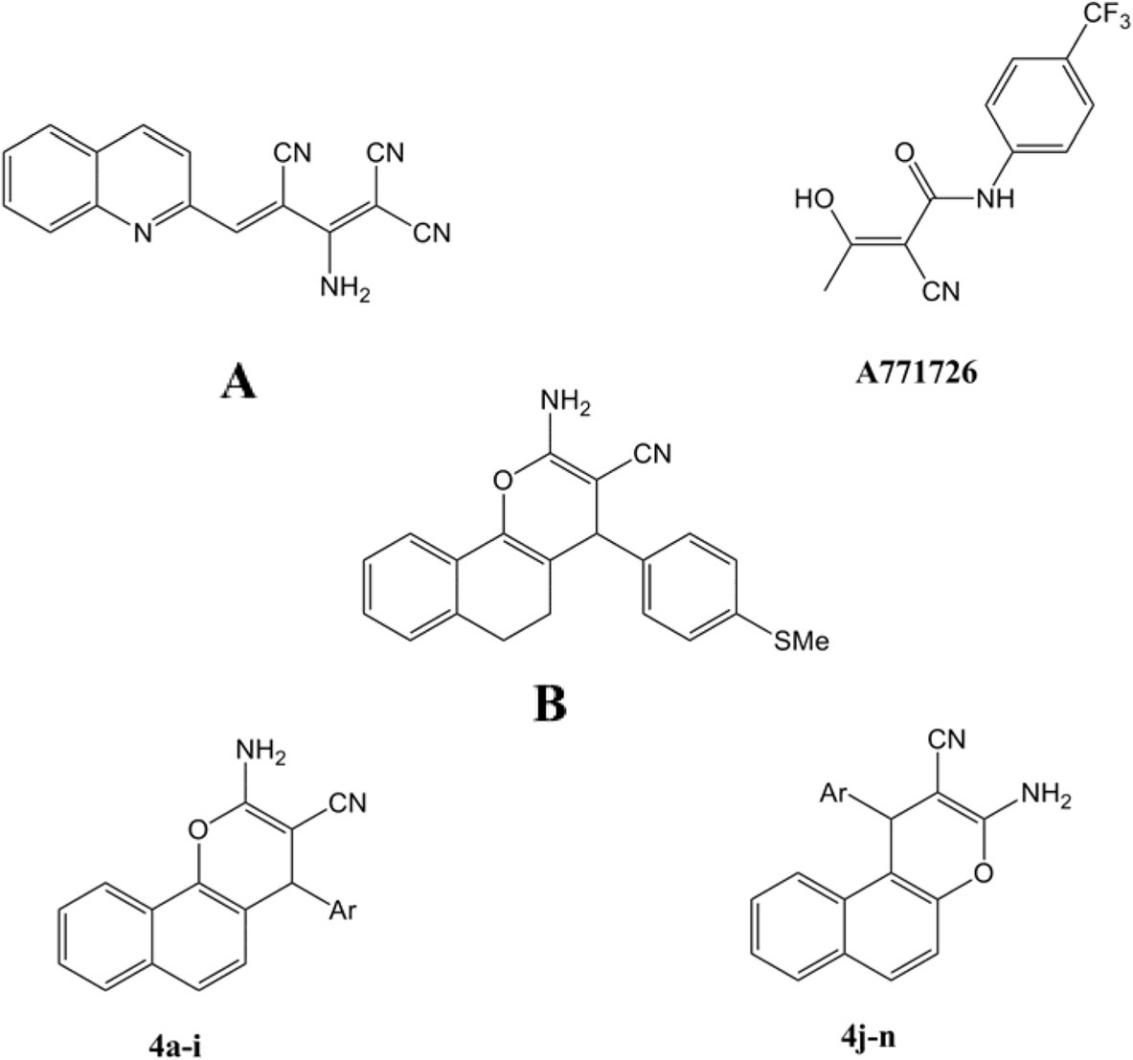
**Benzylidene-malononitrile(tyrophstin) derivative as protein kinase inhibitor (A), Chemical structures of A771726, active metabolite of Leflunomide, antiproliferative 4-aryl-4*H*-5,6-dihydronaphtho[1,2-b]pyran (compound B), and synthesized 4-Aryl-4*H*-Naphthopyrans (4a-n).**

Recently emerging data support the hypothesis that the presence of a nitrile group in different scaffolds, such as quinolinecarbonitrile (e.g., Bosutinib), and benzylidene-malononitriles (tyrophostins (acronym of tyrosine phosphorylation inhibitors)), is crucial for protein kinase inhibitory activity [22]. Compound **A** (Figure 2) [23] with 2-quinoline tyrophostin structure exhibit IC_50_ in the low molecular range (IC_50_= 1.7 μM) against EFGR. Furthermore, A771726 (Figure 2) with cyano hydroxyl β-butenamide, which is the predominant active metabolite of Leflunamide, has been identified as a tyrosine kinase inhibitor and antiproliferative agent [24,25]. This compound was used as a starting lead for designing new antiproliferative compounds that finally led to the identification of 4-aryl-4*H*-5,6-dihydronaphtho[1,2-b]pyran **B** (Figure 2) as a new lead compound with moderate activity on the mesenchymal stem cell proliferation assay (IC_50_ = 13 μM) [25]. Different derivatives of naphthopyranes have been synthesized since then and showed multiple pharmacological effects, such as anti-rheumatoid, anti-diabetic, anti-proliferative, and protease inhibition effects [25,26].

We previously reported synthesis and evaluation of 4-aryl-4*H*-chromene-3-carbonitrile derivatives as Src kinase inhibitors [27]. As part of our efforts to identify new scaffolds as Src kinase inhibitors [28-35], herein we report the synthesis and evaluation of selected 2 or 3-amino-4-aryl-4*H*-benzo[*h* or *f*]chromene-3-carbonitrile derivatives **4a-n** (Figure 2) that contains both nitrile and naphthopyrane cores as Src kinase inhibitors or anti-proliferative agents.

## Methods

### Materials

All starting materials, reagents, and solvents were purchased from Merck AG (Germany). The purity of the synthesized compounds was confirmed by thin layer chromatography (TLC) using various solvents of different polarities. Merck silica gel 60 F254 plates were applied for analytical TLC. Column chromatography was performed on Merck silica gel (70–230 mesh) for purification of the intermediate and final compounds. Melting points were determined on a Kofler hot stage apparatus (Vienna, Austria) and are uncorrected. ^1^H NMR spectra were recorded using a Bruker 500 MHz spectrometer (Bruker, Rheinstatten, Germany), and chemical shifts were expressed as δ (ppm) with tetramethylsilane (TMS) as an internal standard. The IR spectra were obtained on a Shimadzu 470 (Shimadzu, Tokyo, Japan) spectrophotometer (potassium bromide disks). The mass spectra were run on a Finnigan TSQ-70 spectrometer (Thermo Finnigan, West Chester, OH, USA) at 70 eV. Elemental analyses were carried out on a CHN-O-rapid elemental analyzer (Heraeus GmbH, Hanau, Germany) for C, H, and N, and the results were within ± 0.4% of the theoretical values.

### General procedure for the peparation of 2-amino-4-aryl-4*H*-naphthopyran-3-carbonitrile derivatives 4a-n

In general, diammonium hydrogen phosphate (DAHR, 0.5 mmol) was added to a mixture of α or β-naphtol (5 mmol), substituted aryl-aldehyde (5 mmol), and malonitrile (5 mmol) in ethanol (10 mL) and water (10 mL). The reaction mixture was stirred at room temperature for 4 h. After cooling, the precipitated solid was filtered, washed with cold ethanol, and crystallized from the same solvent. The synthesized compounds were characterized by IR, ^1^H NMR, mass spectrpscopy and elemental analysis. Physicochemical and spectral properties of compounds **4a-n** were consistent with the previously reported data [36,37].

### c-Src kinase activity assay

The effect of synthesized compounds on the activity of c-Src kinase was determined by HTScan Src Kinase Assay Kit, catalogue number 7776 from Cell Signaling Technology (Danvers, MA, USA); according to manufacturer’s protocol. Streptavidin-coated plates were purchased from Pierce (Rockford, IL, USA). In brief, the kinase reaction was started with the incubation of the 12.5 μL of the reaction cocktail (0.5 ng/μL of GST-Src kinase in 1.25 mM DTT) with 12.5 μL of prediluted compounds (dissolved in 1% DMSO) for 5 min at room temperature. ATP/substrate (25 μL, 20 μM/1.5 uM) cocktail was added to the mixture. The biotinylated substrate (catalogue number 1366) contains the residues surrounding tyrosine 160 (Tyr160) of signal transduction protein and has a sequence of EGIYDVP. The reaction mixture was incubated for 30 min at room temperature. The kinase reaction was stopped with the addition of 50 μL of 50 mM EDTA (pH 8.0). The reaction solution (25 μL) was transferred into 96-well streptavidin plates (Pierce, part number 15125), diluted with 75 μL double distilled water, and incubated at room temperature for 60 min. At the end of the incubation, the wells were washed three times with 200 μL of 0.05% Tween-20 in PBS buffer (PBS/T). After that to each well was added 100 μL of phosphotyrosine antibody (P-Tyr-100) (1:1000 dilution in PBS/T with 1% BSA) and the wells were incubated for another 60 min. After washing three times with 0.05% Tween-20 in PBS/T, the wells were incubated with 100 μL secondary anti-mouse IgG antibody, which was HRP-conjugated (1:500 dilution in PBS/T with 1% BSA) for next 30 min at room temperature. The wells were washed five times with 0.05% Tween-20 in PBS and then were incubated with 100 μL of 3,3',5,5'-tetramethylbenzidine dihydrochloride (TMB) substrate for 5 min. The reaction was stopped by adding 100 μL/well of stop solution to each well and mixed well and read the absorbance at 450 nm using a microplate reader (Molecular devices, spectra Max M2). IC_50_ values of the compounds were calculated using ORIGIN 6.0 (origin lab) software. IC_50_ is the concentration of the compound that inhibited enzyme activity by 50%. All the experiments were carried out in triplicate [27].

### Cell culture and cell proliferation assay

#### Cell culture

Breast carcinoma BT-20 (ATCC no. HTB-19) cell lines were obtained from American Type Culture Collection. Cells were grown on 75 cm^2^ cell culture with EMEM medium for breast carcinoma and supplemented with 10% fetal bovine serum (FBS), and 1% penicillin-streptomycin solution (10,000 units of penicillin and 10 mg of streptomycin in 0.9% NaCl) in a humidified atmosphere of 5% CO_2_, 95% air at 37°C.

### Cell proliferation assay

Cell proliferation assay of compounds was evaluated in BT-20 cells, and was compared with that of doxorubicin (DOX)[38]. Cell proliferation assay was carried out using CellTiter 96 aqueous one solution cell proliferation assay kit (Promega, USA). Briefly, upon reaching about 75-80% confluence, 5000 cells/well were plated in 96-well microplate in 100 μL media. After seeding for 72 h, the cells were treated with 50 μM compound in triplicate. DOX (10 μM) was used as the positive control. Incubation was carried out at 37°C in an incubator supplied with 5% CO_2_ for 72 h. At the end of the sample exposure period (72 h), 20 μL CellTiter 96 aqueous solution was added. The plate was returned to the incubator for 1 h in a humidified atmosphere at 37°C. The absorbance of the formazan product was measured at 490 nm using microplate reader. The blank control was recorded by measuring the absorbance at 490 nm with wells containing medium mixed with CellTiter 96 aqueous solution but no cells. Results were expressed as the percentage of the control (DMSO without compound set at 100%). The percentage of cell survival was calculated as (OD value of cells treated with test compound - OD value of culture medium) / (OD value of control cells – OD value of culture medium) × 100%.

## Results and discussion

### Chemistry

**Figure 3:**
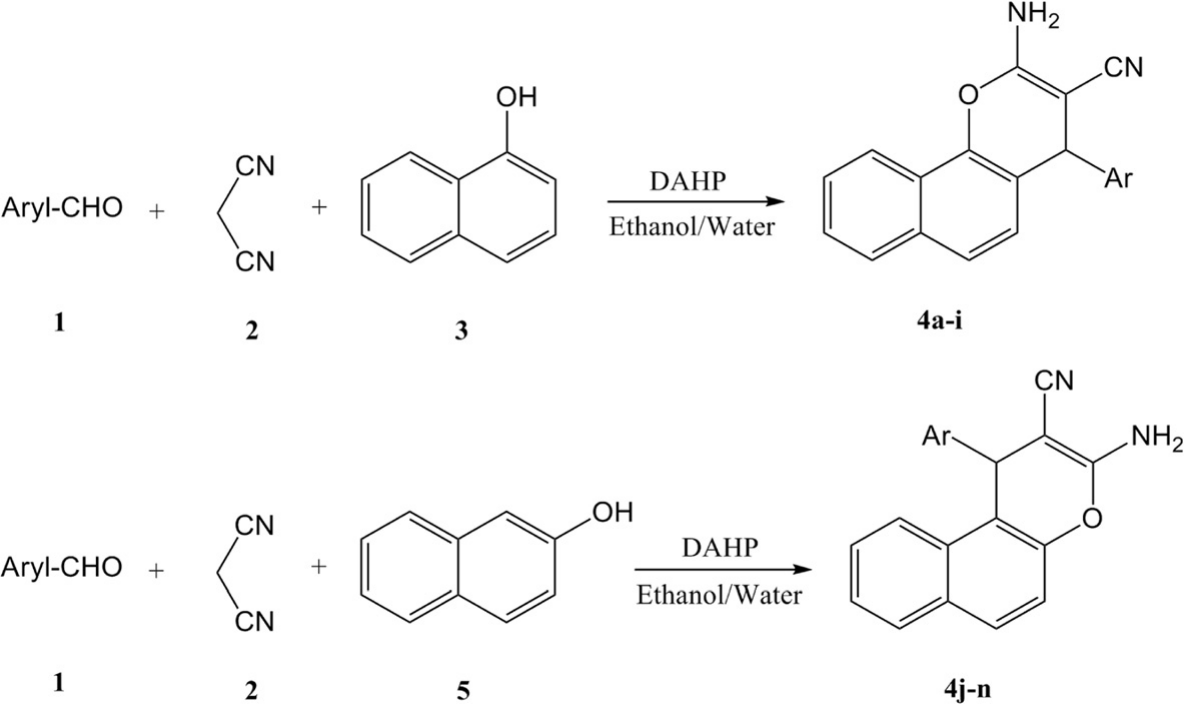
**Inhibition of BT-20 cell proliferation by compounds 4a-n (50 μM) after 72 h incubation. **The results are shown as the percentage of the control DMSO that has no compound (set at 100%). All the experiments were performed in triplicate.

**Figure 4:**
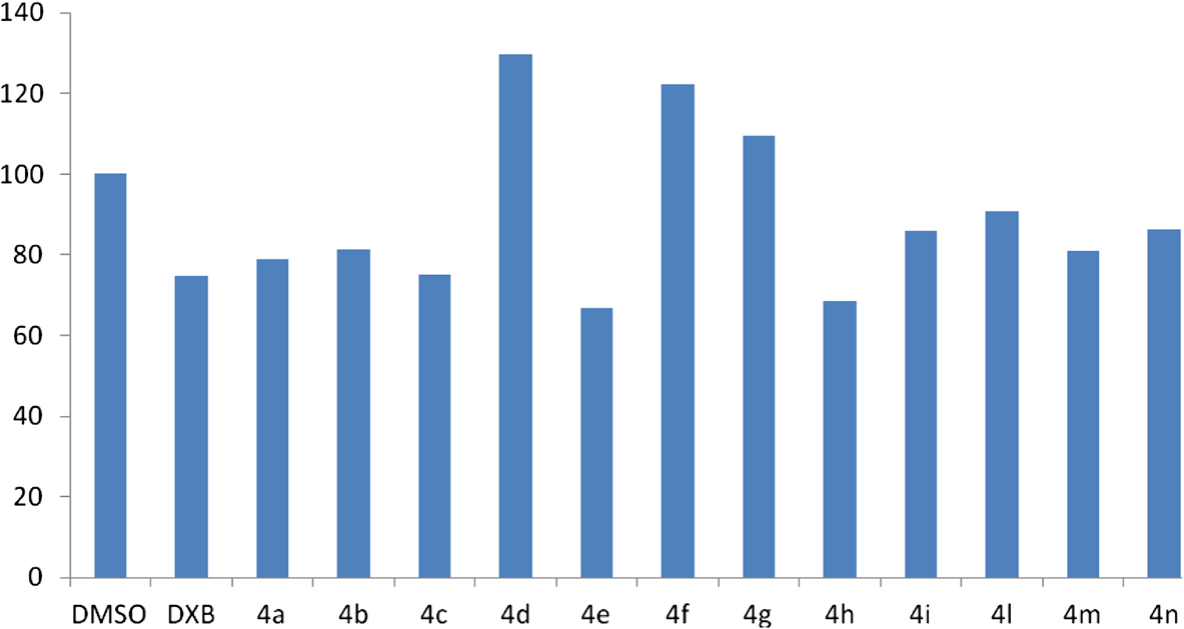
**One-pot synthesis of 4-Aryl-4*H*-Naphthopyrans 4a-n.**

Naphthopyran analogs described here (**4a-n**) were synthesized following the synthetic routes outlined in Figure 3. The compounds were synthesized according to the previously reported general procedure by a one-pot reaction [37] through the condensation of a substituted aromatic aldehyde (**1**), malonitrile, and α or β-naphtol (**3, 5**) in ethanol and water in the presence of diammonium hydrogen phosphate (Figure 4).

### Src kinase inhibitory activity

The results of Src kinase inhibitory activity of compounds **4a-n** are shown in Table 1. Compounds **4a**, **4d**, **4i**, **4m**, and **4n** exhibited higher Src kinase inhibitory activity (IC_50_ =28.1– 34.7 μM in following order **4a**>**4i,4m**>**4n**>**4d**) when compared with that of other compounds. Unsubstituted phenyl analog **4a** showed an IC_50_ value of 28.1 μM and was the most potent compound in this series. 2-Chlorophenyl substituted analog **4d** was one of the potent compounds in α-naphthol series with an IC_50_ value of 34.7 μM. Similarly compound **4j** with 3-chlorophenyl substitution was a moderately effective compound in β-naphthol series with an IC_50_ value of 41.7 μM. In addition, 2,3-dichlorophenyl substituted compounds **4f** and **4l** were among potent inhibitors in both α and β-naphthol series of compounds. However, substitution of 2,6-dichlorophenyl in compound **4g** led to loss of inhibitory activity, suggesting that chloro substitution at positions 2 and 6 of phenyl ring is detrimental in Src kinase inhibition. Compounds **4m** and **4n** with 3-hydroxyphenyl and 4-methoxyphenyl moieties, respectively, were among potent compounds in β-naphthol series. 3-Nitrophenyl substitution resulted in loss of activity in compounds **4e** and **4k** in both α and β-naphthol series of compounds**.** Similarly compounds **4b** (4-F-Ph) and **4c** (3-Br-Ph) did not show any significant inhibitory activity (IC_50_ > 150) against Src kinase. On the other hand, compounds **4h** with pyridine ring substitution and compound **4i** with 1-methylnitroimidazole moiety showed good inhibitory activity against Src kinase compared to other phenyl substituted compounds.

**Table 1:** **Inhibition of Src Kinase activity by 2-amino-4-aryl-4 *H *-naphtopyran-3-carbonitrile derivatives**


**Compounds**	**Ar**	**IC**_**50 **_**(μM)**^**a**^
**4a**	Ph	28.1
**4b**	4-F-Ph	> 150
**4c**	3-Br-Ph	> 150
**4d**	2-Cl-Ph	34.7
**4e**	3-NO_2_-Ph	> 150
**4f**	2,3-di(Cl)-Ph	41.7
**4g**	2,6-di(Cl)-Ph	> 150
**4h**		36.5
**4i**		33.8
**4j**	3-Cl-Ph	41.7
**4k**	3-NO_2_-Ph	> 150
**4l**	2,3-di(Cl)-Ph	38.9
**4m**	3-OH-Ph	33.8
**4n**	4-MeO-Ph	34.5
Staurosporin	-	0.3
PP2	-	2.8

^a^The concentration that inhibited enzyme activity by 50%.

### Anti-proliferative activities

The effect of the inhibitors at the concentration of 50 μM on the cell proliferation of breast carcinoma (BT-20) cell line was evaluated (Figure 3). Compounds **4e** and **4h** inhibited the cell proliferation of breast carcinoma cells (BT-20) by 33% and 31.5% (**4h**>**4e**), respectively (Figure 3). Compounds **4e** and **4h** found to be more potent on BT-20 cell proliferation assay compared to doxorubicin, while compound **4c** with 24.8% cell proliferation inhibitory effect against BT-20 cell line was as effective as doxorubicin (25% inhibition of cell growth).

Although compound **4h** was found to be a moderate Src kinase inhibitor and a potent anti-proliferative agent against BT-20 cell line, there were no significant correlation between Src kinase inhibitory potency of the other compounds with their growth inhibition activity against cancer cells. Compounds **4a**, **4d**, **4i**, **4m**, and **4n** with good Src kinase inhibitory activity showed less potency in anti-proliferation assay (0-21%). In contrast, compound **4e** that showed good anti-proliferative effect against breast carcinoma cells was a weak Src kinase inhibitor (IC_50_ > 150 μM). It seems that *in vitro* enzymatic and cell-based assays may not correlate well due to the diversity in solubility and cellular uptake of the compounds. Further studies are required to determine whether these compounds have limited cellular uptakes that lead to their minimal anti-proliferative activities.

## Conclusions

A number of 2-amino-4-aryl-4*H*-benzo[h or f]chromene-3-carbonitrile derivatives were prepared and evaluated for Src kinase inhibitory and anticancer activities. In summary, structure-activity relationship studies revealed that the incorporation of less bulkier groups such as unsubstituted phenyl at position of aryl is preferred and well tolerated compared to other groups in generating Src inhibitory activity. The data provide insights for key structural requirements for further optimization of chromene-carbonitrile derivatives as a scaffold and generating more potent and selective Src kinase inhibitors and/or anticancer agents.

## Competing interest

The authors declare that there is no conflict of interest related to this publication.

## Authors’ contributions

AR contributed to the synthesis of some target compounds. AFT contributed to synthesis of the intermediates and some target compounds, and preparation of the manuscript. RK participated in evaluation of the Src Kinase Inhibitory activities. ANS and DM participated in evaluation of the anti-proliferative activities. KP collaborated in management of the pharmacological part, and edition of manuscript. AS contributed in identifying of the structures of target compounds. AF collaborated in design of target compounds. TA participated in designing of target compounds, management of the synthetic and pharmacological parts, and preparation of the manuscript and approval of final article. All authors read and approved the final manuscript.

## References

[B1] Yeatman TJ A renaissance for SRC Nat Rev Cancer 2004 4 470 480 10.1038/nrc1366 15170449

[B2] Yu CL Meyer DJ Campbell GS Larner AC Carter-Su C Schwartz J Jove R Enhanced DNA-binding activity of a Stat3-related protein in cells transformed by the Src oncoprotein Science 1995 269 81 83 10.1126/science.7541555 7541555

[B3] Niu G Wright KL Huang M Song L Haura E Turkson J Zhang S Wang T Sinibaldi D Coppola D Heller R Ellis LM Karras J Bromberg J Pardoll D Jove R Yu H Constitutive Stat3 activity up-regulates VEGF expression and tumor angiogenesis Oncogene 2002 21 2000 2008 10.1038/sj.onc.1205260 11960372

[B4] Russello SV Shore SK SRC in human carcinogenesis Front Biosci 2004 9 139 144 14766353 10.2741/1138

[B5] Tsygankov AY Shore SK Src: regulation, role in human carcinogenesis and pharmacological inhibitors Curr Pharm Des 2004 10 1745 1756 10.2174/1381612043384457 15180537

[B6] Boyer B Valles AM Edme N Induction and regulation of epithelial-mesenchymal transitions Biochem Pharmacol 2000 60 1091 1099 10.1016/S0006-2952(00)00427-5 11007946

[B7] Johnson FM Gallick GE SRC family nonreceptor tyrosine kinases as molecular targets for cancer therapy Anticancer Agents Med Chem 2007 7 651 659 10.2174/187152007784111278 18045060

[B8] Nam JS Ino Y Sakamoto M Hirohashi S Src family kinase inhibitor PP2 restores the E-cadherin/catenin cell adhesion system in human cancer cells and reduces cancer metastasis Clin Cancer Res 2002 8 2430 2436 12114449

[B9] Coluccia AM Benati D Dekhil H De Filippo A Lan C Gambacorti-Passerini C SKI-606 decreases growth and motility of colorectal cancer cells by preventing pp 60(c-Src)-dependent tyrosine phosphorylation of beta-catenin and its nuclear signaling Cancer Res 2006 66 2279 2286 10.1158/0008-5472.CAN-05-2057 16489032

[B10] Gonzalez L Agullo-Ortuno MT Garcia-Martinez JM Calcabrini A Gamallo C Palacios J Aranda A Martin-Perez J Role of c-Src in human MCF7 breast cancer cell tumorigenesis J Biol Chem 2006 281 20851 20864 10.1074/jbc.M601570200 16728403

[B11] Benati D Baldari CT SRC family kinases as potential therapeutic targets for malignancies and immunological disorders Curr Med Chem 2008 15 1154 1165 10.2174/092986708784310404 18473810

[B12] Sundaramoorthi R Shakespeare WC Keenan TP Metcalf CA III Wang Y Mani U Taylor M Liu S Bohacek RS Narula SS Dalgarno DC van Schravandijk MR Violette SM Liou S Adams S Ram MK Keats JA Weigle M Sawyer TK Weigele M Bone-targeted Src kinase inhibitors: novel pyrrolo- and pyrazolopyrimidine analogues Bioorg Med Chem Lett 2003 13 3063 3066 10.1016/S0960-894X(03)00647-4 12941334

[B13] Schenone S Bruno O Ranise A Bondavalli F Brullo C Fossa P Mosti L Menozzi G Carraro F Naldini A Bernini C Manetti F Botta M New pyrazolo[3,4-d]pyrimidines endowed with A431 antiproliferative activity and inhibitory properties of Src phosphorylation Bioorg Med Chem Lett 2004 14 2511 2517 10.1016/j.bmcl.2004.03.013 15109642

[B14] Ple PA Green TP Hennequin LF Curwen J Fennell M Allen J Lambert-Van Der Brempt C Costello G Discovery of a new class of anilinoquinazoline inhibitors with high affinity and specificity for the tyrosine kinase domain of c-Src J Med Chem 2004 47 871 887 10.1021/jm030317k 14761189

[B15] Hennequin LF Allen J Breed J Curwen J Fennell M Green TP Lambert-van Der Brempt C Morgentin R Norman RA Olivier A Otterbein L Ple PA Warin N Costello G N-(5-chloro-1,3-benzodioxol-4-yl)-7-[2-(4-methylpiperazin-1-yl)ethoxy]-5- (tetrahydro-2H-pyran-4-yloxy)quinazolin-4-amine, a novel, highly selective, orally available, dual-specific c-Src/Abl kinase inhibitor J Med Chem 2006 49 6465 6488 10.1021/jm060434q 17064066

[B16] Barrios Sosa AC Boschelli DH Wu B Wang Y Golas JM Further studies on ethenyl and ethynyl-4-phenylamino-3-quinolinecarbonitriles: identification of a subnanomolar Src kinase inhibitor Bioorg Med Chem Lett 2005 15 1743 1747 10.1016/j.bmcl.2005.01.004 15745832

[B17] Boschelli DH Wu B Ye F Durutlic H Golas JM Lucas J Boschelli F Facile preparation of new 4-phenylamino-3-quinolinecarbonitrile Src kinase inhibitors via 7-fluoro intermediates: identification of potent 7-amino analogs Bioorg Med Chem 2008 16 405 412 10.1016/j.bmc.2007.09.028 17905586

[B18] Boschelli DH Wang D Wang Y Wu B Honores EE Barrios Sosa AC Chaudhary I Golas J Lucas J Boschelli F Optimization of 7-alkene-3-quinolinecarbonitriles as Src kinase inhibitors Bioorg Med Chem Lett 2010 20 2924 2927 10.1016/j.bmcl.2010.03.025 20363128

[B19] Dulsat C Mealy N Castaner R Saracatinib. Dual Src/ABL kinase inhibitor, Oncolytic Drugs Fut 2009 34 106 114 10.1358/dof.2009.034.02.1340647

[B20] Boschelli DH Ye F Wang YD Dutia M Johnson SL Wu B Miller K Powell DW Yaczko D Young M Tischler M Arndt K Discafani C Etienne C Gibbons J Grod J Lucas J Weber JM Boschelli F Optimization of 4-phenylamino-3-quinolinecarbonitriles as potent inhibitors of Src kinase activity J Med Chem 2001 44 3965 3977 10.1021/jm0102250 11689083

[B21] Boschelli DH Boschelli F Bosutinib. Dual Src and Abl kinase inhibitor, treatment of solid tumors, treatment of CML and Ph+ ALL Drugs Fut 2007 32 481 490 10.1358/dof.2007.032.06.1107656

[B22] Lawrence DS Niu J Protein kinase inhibitors: the tyrosine-specific protein kinases Pharmacol Ther 1998 77 81 114 10.1016/S0163-7258(97)00052-1 9578319

[B23] Brunton VG Lear MJ McKeown P Robins DJ Workman P Synthesis and antiproliferative activity of tyrphostins containing quinoline moieties Anticancer Drug Des 1996 11 463 483 8836111

[B24] Morin MJ From oncogene to drug: development of small molecule tyrosine kinase inhibitors as anti-tumor and anti-angiogenic agents Oncogene 2000 19 6574 6583 10.1038/sj.onc.1204102 11426642

[B25] Dell CP Antiproliferative naphthopyrans: biological activity, mechanistic studies and therapeutic potential Current Med Chem 1998 5 179 194 9562601

[B26] Wiernicki TR Bean JS Dell C Williams A Wood D Kauffman RF Singh JP Inhibition of vascular smooth muscle cell proliferation and arterial intimal thickening by a novel antiproliferative naphthopyran J Pharmacol Exp Ther 1996 278 1452 1459 8819533

[B27] Fallah-Tafti A Tiwari R Shirazi AN Akbarzadeh T Mandal D Shafiee A Parang K Foroumadi A 4-aryl-4H-chromene-3-carbonitrile derivatives: evaluation of Src kinase inhibitory and anticancer activities Med Chem 2011 7 466 472 10.2174/157340611796799258 21801146

[B28] Fallah-Tafti A Foroumadi A Tiwari R Shirazi AN Hangauer DG Bu Y Akbarzadeh T Parang K Shafiee A Thiazolyl N-benzyl-substituted acetamide derivatives: synthesis, Src kinase inhibitory and anticancer activities Eur J Med Chem 2011 46 4853 4858 10.1016/j.ejmech.2011.07.050 21852023

[B29] Kumar D Reddy VB Kumar A Mandal D Tiwari R Parang K Click chemistry inspired one-pot synthesis of 1,4-disubstituted 1,2,3-triazoles and their Src kinase inhibitory activity Bioorg Med Chem Lett 2011 21 449 452 10.1016/j.bmcl.2010.10.121 21084189

[B30] Sharma D Sharma RK Bhatia S Tiwari R Mandal D Lehmann J Parang K Olsen CE Parmar VS Prasad AK Synthesis, Src kinase inhibitory and anticancer activities of 1-substituted 3-(N-alkyl-N-phenylamino)propane-2-ols Biochimie 2010 92 1164 1172 10.1016/j.biochi.2010.04.022 20447438

[B31] Tiwari R Brown A Narramaneni S Sun G Parang K Synthesis and evaluation of conformationally constrained peptide analogues as the Src SH3 domain binding ligands Biochimie 2010 92 1153 1163 10.1016/j.biochi.2010.01.017 20109515

[B32] Kumar A Wang Y Lin X Sun G Parang K Synthesis and evaluation of 3-phenylpyrazolo[3,4-d]pyrimidine-peptide conjugates as Src kinase inhibitors Chem Med Chem 2007 2 1346 1360 17530729 10.1002/cmdc.200700074

[B33] Kumar A Ye G Wang Y Lin X Sun G Parang K Synthesis and structure-activity relationships of linear and conformationally constrained peptide analogues of CIYKYY as Src tyrosine kinase inhibitors J Med Chem 2006 49 3395 3401 10.1021/jm060334k 16722659 PMC2527579

[B34] Nam NH Lee S Ye G Sun G Parang K ATP-phosphopeptide conjugates as inhibitors of Src tyrosine kinases Bioorg Med Chem 2004 12 5753 5766 10.1016/j.bmc.2004.08.043 15498652

[B35] Nam NH Ye G Sun G Parang K Conformationally constrained peptide analogues of pTyr-Glu-Glu-Ile as inhibitors of the Src SH2 domain binding J Med Chem 2004 47 3131 3141 10.1021/jm040008+ 15163193

[B36] Balalaie S Ramezanpour S Bararjanian M Gross JH DABCO‐catalyzed efficient synthesis of naphthopyran derivatives via One‐Pot three‐component condensation reaction at room temperature Synthetic Commun 2008 38 1078 1089 10.1080/00397910701862865

[B37] Abdolmohammadi S Balalaie S Novel and efficient catalysts for the one-pot synthesis of 3,4-dihydropyrano[c]chromene derivatives in aqueous media Tetrahedron Lett 2007 48 3299 3303 10.1016/j.tetlet.2007.02.135

[B38] Chhikara BS St Jean N Mandal D Kumar A Parang K Fatty acyl amide derivatives of doxorubicin: synthesis and in vitro anticancer activities Eur J Med Chem 2011 46 2037 2042 10.1016/j.ejmech.2011.02.056 21420207

